# In respond to commensal bacteria: γδT cells play a pleiotropic role in tumor immunity

**DOI:** 10.1186/s13578-021-00565-w

**Published:** 2021-03-02

**Authors:** Yongting Liu, Ying Han, Shan Zeng, Hong Shen

**Affiliations:** 1 Department of Oncology, Xiangya Hospital, Central South University, Changsha, 410008 Hunan China; 2 National Clinical Research Center for Geriatric Disorders, Xiangya Hospital, Central South University, Changsha, 410008 Hunan P.R. China; 3 Key Laboratory for Molecular Radiation Oncology of Hunan Province, Xiangya Hospital, Central South University, Changsha, 410008 Hunan China

**Keywords:** Γδ T cells, Commensal bacteria, Protumor effects, Antitumor effects, Immunity therapy

## Abstract

γδT cells are a mixture of innate programming and acquired adaptability that bridge the adaptive and innate immune systems. γδT cells are mainly classified as tissue-resident Vδ1 or circulating Vδ2 γδT cells. In the tumor microenvironment, tumor immunity is influenced by the increased quantity and phenotype plasticity of γδT cells. Commensal bacteria are ubiquitous in the human body, and they have been confirmed to exist in various tumor tissues. With the participation of commensal bacteria, γδT cells maintain homeostasis and are activated to affect the development and progression of tumors. Here, we summarize the relationship between γδT cells and commensal bacteria, the potential protumor and antitumor effects underlying γδT cells, and the new developments in γδT cell-based tumor therapy which is expected to open new opportunities for tumor immunotherapy.

## Background

### Commensal bacteria

The human body comprises 10% cells and 90% bacteria. These bacteria reside in the skin, gastrointestinal tract, breast, lung, urinary tract and other regions. They are collectively known as commensal bacteria and have a complex connection with tumor immunity in the body [1]. With the latest developments in 16S rRNA gene sequencing and metagenomic analysis, an increasing number of bacteria have been found in the tumor microenvironment (TME). In an experiment with more than 300 samples of 7 solid tumors the distribution of bacteria was tissue-specific and tumor sub-type specific. One thing in common was that Proteobacteria and Firmicutes phyla account for most of the detected bacterial species, but the ratio of Proteobacteria to Firmicutes seems to vary between tumor types [2].

Cancer patients experience an imbalanced microbiota state called "dysbiosis", which is reflected in a substantial reduction in bacterial diversity and community stability [3]. Independent of specific bacterial species, dysbiosis promotes the development and progression of tumors [4]. It is mediated by a decrease in tumor necrosis factor alpha (TNF-α) levels in tissues and the blood circulation, leading to reduced expression of tumor endothelial adhesion molecules, especially intercellular adhesion molecule 1 (ICAM-1). The expression of ICAM-1 is reduced by more than 50%, ultimately reducing the antitumor effect of CD8^+^ T cells [5, 6]. Dysbiosis also affects the response to chemotherapy, including the traditional chemotherapeutic drug cyclophosphamide [7] and new immune checkpoint inhibitors [8, 9].

### γδT cells

γδT cells are a mixture of innate programming and acquired adaptability that bridge the adaptive and innate immune systems [10, 11]. They are mainly distributed in the skin and mucosal epithelium and account for the majority of tissue-resident T cells. In addition, 1–5% of γδ T cells are found in peripheral blood [12]. In terms of TCRδ chain usage, Vδ1T cells are mainly located in the skin and mucous membranes and interact with Vγ2, γ3, γ4, γ5 and γ8 chains to maintain epithelial stability. Vδ2Vγ9T cells account for up to 90% of circulating γδT cells and can be recruited to the corresponding tissues to perform their functions [13].

It is worth noting that there are some species heterogeneities in gene evolution of TCR between humans and mice. Mice γδT cells depends on the specific TCR Vγ chain, Vγ 1–7. In spite of the discrepancy, γδT cells have functional similarity in mice and humans [14].

Most γδT cells are CD4^−^ and CD8^−^ cells, and their antigen recognition is not subject to major histocompatibility complex (MHC) restriction. γδT cells can also be activated by cytokines independent of their γδTCRs and take effect earlier. The activation, expansion, migration and functional plasticity of intratumor γδT cells are driven by changes in the TME, and these properties have a significant impact on maintaining mucosal stability and tumor immunity [13, 15].

Here, we review the relationship between commensal bacteria and γδT cells as well as the mechanism behind the dual effects of γδT cells on tumors harboring commensal bacteria. These findings are expected to identify new targets for tumor immunotherapy.

### Commensal bacteria participate in the homeostasis of γδT cells

The homeostasis of γδT cells is affected by commensal bacteria. In dysbiosis or germ-free (GF) mice, the number of γδT cells is significantly reduced compared to that in their conventionally housed, specific pathogen-free (SPF) counterparts, as confirmed in the liver, lungs, intestines and peritoneum [16, 17]. The intestinal mucosa can be used as an example. Under normal circumstances, γδT cells rely on the communication between aryl hydrocarbon receptors (AhRs) and interleukin (IL)-15 produced by intestinal epithelial cells stimulated by microorganisms [18]. In GF models, Bifidobacteriaceae and Bacillaceae are positively related to intestinal γδT cells, while bacteria belonging to the families Rhodospirillaceae, Flavobacteriaceae and Prevotellaceae have the opposite relationship [16]. In the liver, *Escherichia coli* (*E. coli*) transplantation can improve γδT cell deficiency, but the *Escherichia coli* population is not irreplaceable [19]. Intraperitoneal injection of neomycin sulfate and vancomycin to kill facultative gram-positive and/or gram-negative organisms may result in lower numbers of γδT cells in the peritoneum of the treated group than the control group. However, metronidazole treatment has no effect on the number of γδT cells [20].

In general, there are few identified bacteria that are particularly relevant to γδT cells. In any case, stability of the commensal bacterial population is important for the homeostasis of γδT cells.

### Commensal bacteria activate γδT cells via different mechanisms

The binding of bacterial pathogen-associated molecular patterns (PAMPs) to Toll-like receptors (TLRs) on γδT cells exerts an activating effect through the myeloid differentiation factor 88 (MyD88) pathway [21]. Although the study on TLRs of human γδT cells is not sufficient, the current unified conclusion is that γδT cells have TLR1 ~ 8 [21, 22]. The TLR2 and TLR5 can recognize lipopolysaccharide and flagellin, perceiving commensal bacteria. TLR3 mainly cooperates with TCR to play an antiviral effect [23]. The activation of TLR8 can reverse the immunosuppressive function of γδT cells [24]. Other TLRs are poorly expressed and rarely studied. Moreover, phagocytes produce IL-1, an inflammatory factor whose production is stimulated by commensal bacteria. IL-1 can be recognized by γδT cells and function through an IL-1R-Vav guanine nucleotide exchange factor 1 (VAV1)-dependent mechanism [20]. Vδ1 TCR has a special affinity for CD1-presented lipid sulfatide, modulated by the complementarity-determining region 3 loop to discriminate different lipid antigens, especially intestinal γδT cells [25, 26]. Another study confirmed that γδT cells in the liver but not the spleen are uniquely sensitive to lipid antigens derived from *E. coli* [27]. Phosphoantigens, such as bacterial lysate-derived (E)-4-hydroxy-3-methyl-but-2-enyl pyrophosphate (HMBPP), are also powerful stimulators of γδT cells [28]. As the most potent phosphoantigen known to stimulate γδT cells, HMBPP mainly activates circulating Vδ2Vγ9T cells [29]. HMBPP binding to intracellular domain of butyrophilin 3A1 (BTN3A1) leads to the extracellular detection by the Vδ2Vγ9 TCR, which reinforces the efficiency of γδT cell activation [30, 31] (Table 1).

**Table 1:** γδT cells activation: ligands and receptors

Source	Ligand	Receptor	References
Bacterial	PAMP	TLR2/5	[22]
	IL-1	IL-1R	[20]
	Lipid antigen-CD1d	Vδ1TCR	[25, 26]
	HMBPP-BTN3A1	Vδ2TCR	[30, 92]
Cancer cell	MICA/B, ULBP	NKG2D	[55, 57]
	hMSH2	NKG2D	[64]
	Nectin-like 5	DNAM-1	[54]
	B7-H6	NKp30	[59, 60]
	Not known	NKp46	[58]
	F1-ATPase	Vδ2TCR	[61]
	annexin A2	Vδ3TCR	[62]
	IPP-BTN3A1	Vδ2TCR	[81, 84]

### Commensal bacteria play an important role in the migration of γδT cells

Similar to αβT cells, γδT cells are highly dynamic. Both tissue-resident and circulating γδT cells can rapidly migrate and be recruited to the effector site. Many studies have focused on gut-resident cells. Epithelium-mediated microbial sensing is part of an important mechanism [32]. γδT cells actively respond to bacterial signals and migrate from the basal to the apical surface of the epithelium, which is in direct contact with bacteria. This process occurs via a mechanism dependent on occluding, which is expressed by γδT cells [33, 34]. IL-15 is also engaged in that mechanism [35]. Vertical displacement of γδT cells is reduced in mice devoid of a microbiota [36], which reemphasizes the importance of bacteria. In addition, Vδ2T cells highly express C-X-C chemokine receptor 3 (CXCR3), C–C motif chemokine receptor 5 (CCR5) and, to a lesser extent, CCR2, guiding the recruitment of γδT cells from blood to tissues [37].

### Classification of intratumoral γδT cells

Hinging on the TME, γδT cells undergo functional plasticity and differentiate into different phenotypes [38]. The actions of γδT17 cells are indispensable within the tumor and are the main producers of IL-17 in tumor tissues [20, 39, 40]. Unlike mice, there has been some evidence that human γδT17 cells are not pre-programmed in the thymus, but acquire IL-17 expression bias from the periphery under the stimulation of stable commensal bacteria and the participation of many cytokines [41, 42]. Tumor-associated myeloid cells sense microbial stimulation via MyD88 and TIR-domain-containing adaptor inducing interferon-β (TRIF) pathways and secrete IL-1 and IL-23, which are key inducers of γδT17 cells [43, 44]. In addition, IL-7 has been proven to be a more rapidly responsive IL-17 stimulator in solid tumors [45, 46]. IL-7 preferentially activates STAT3 in γδT cells rather than STAT5 in Th17 cells, significantly expanding the γδT17 cells, in both humans and mice [47]. Retinoic acid-related orphan nuclear receptor gamma t (RORγt) is also related to the polarization of γδT17 cells. This characteristic is specifically manifested as the induced expression of the gene encoding IL-17 by RORγt when γδT cells are stimulated with transforming growth factors (TGF-β) and IL-6 [48]. TGF-β also promotes the polarization of Foxp3^+^ regulatory γδT (γδTreg) cells with cooperation from IL-15 in vitro [49, 50]. Cytotoxic helper γδT1 (γδTh1) cells are also found in the TME and their properties are selectively acquired upon stimulation with IL-2 or IL-15 [51, 52] (Table 2).

**Table 2:** The phenotypes, stimulators and effects of intratumoral γδT cells

Phenotype	Stimulator	Effect	References
γδT17	IL-7; IL-1, IL-23; TGF-β, IL-6	Recruit PMN-MDSC and TAN, increase VEGF, express anti-apoptotic genes	[43, 44, 47, 70, 75]
γδTreg	TGF-β, IL-15	Increase adenosine, inhibit γδTh1	[49, 80]
γδTh1	IL-2, IL-15	Secret IFN-γ	[51, 52]
γδT-APC	PAMP	Regulate CD4+ or CD8+ T cells, induce mucosa to release calprotectin	[67, 68]

### The dual effects of γδT cells on tumors

#### Antitumor effect (Fig. 1)

**Fig. 1:**
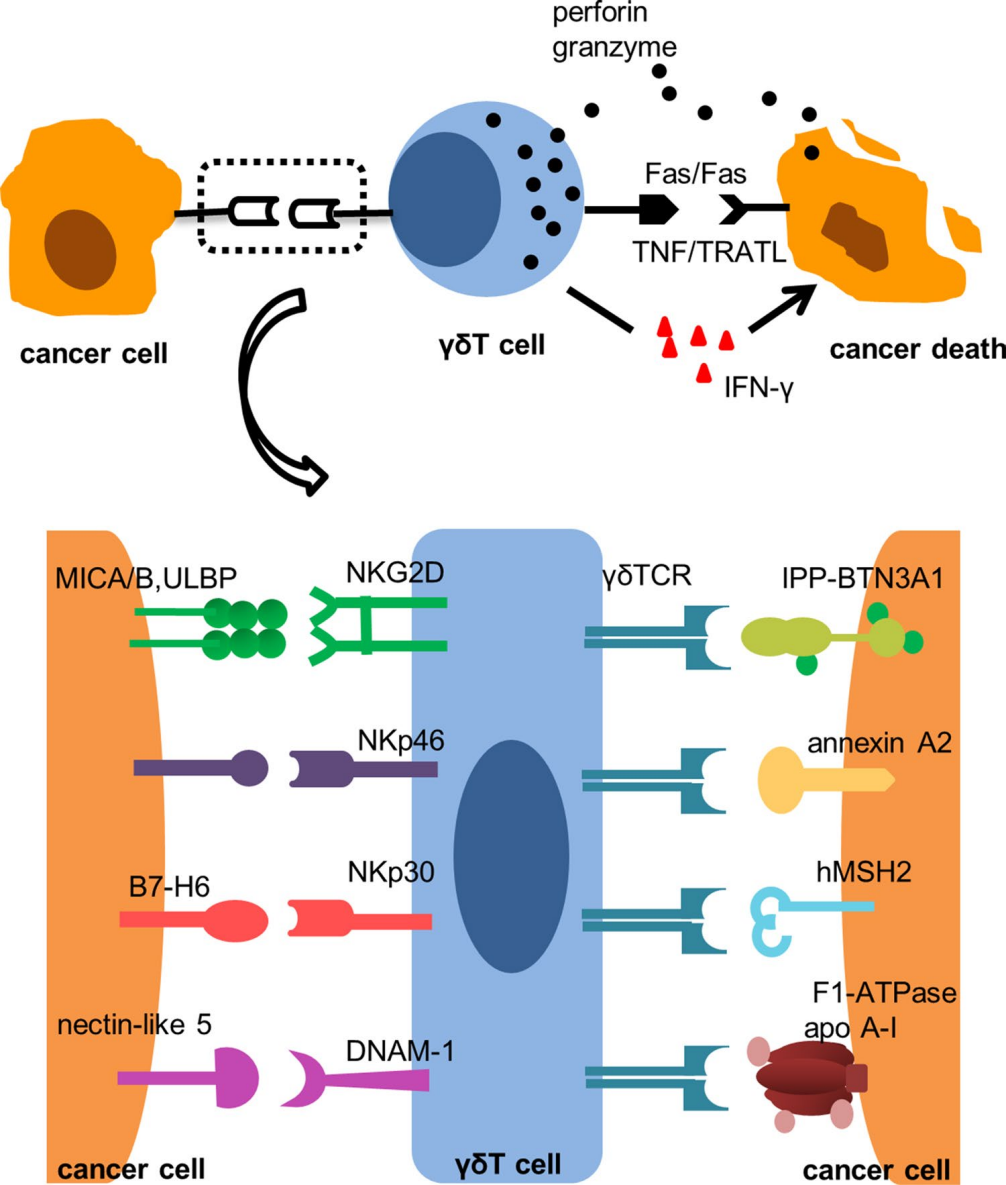
Antitumor effects of γδT cells. γδT cells inhibit tumor growth through receptor-ligand interactions. MHC class I-related chains A/B (MICA/B) and UL16-binding protein (ULBP) are upregulated in cancer cells and are recognized by natural killer, group 2, member D (NKG2D), which exerts cytotoxic effects in cooperation with γδTCR. DNAX accessory molecule-1 (DNAM-1) and nectin-like-5 have been proven to interact on Vδ2Vγ9T cells in hepatocellular carcinoma. Natural cytotoxicity receptors (NCRs), especially NKp46, which is negatively correlated with the risk of metastasis in colorectal cancer, are abundantly expressed on Vδ1T cells. The ligand for NKp30 on Vδ1T cells is B7-H6, which is common in lymphoma and leukemia. Tumor surface protein: hMSH2, F1-ATPase-related structure and annexin A2 could constitute danger signals for γδT cells recognition, reducing the possibility of immune shielding. Like natural killer cells, γδT cells kill cancer cells indirectly by releasing interferon-gamma (IFN-γ), thereby exhibiting a Th1 cell-like phenotype, or directly via the death receptor signal factor associated suicide ligand (FasL) and TNF-related apoptosis-inducing ligand (TRAIL), secreting cytotoxic molecules such as granzyme and perforin

A meta-analysis of 18,000 human tumor samples clarified that intratumoral γδT cells participate in the formation of the most favorable cell population for cancer prognosis [53]. γδT cells express multiple natural killer receptors, including natural killer, group 2, member D (NKG2D); the DNAX accessory molecule-1 (DNAM-1) receptor; and the natural cytotoxicity receptor (NCR) [54–56]. MHC class I-related chains A/B (MICA/B) and UL16-binding proteins (ULBP) are upregulated ligands in cancer cells that are recognized by NKG2D and exert cytotoxic effects in cooperation with γδTCR [55, 57]. DNAM-1 and nectin-like-5 have been demonstrated to interact on Vδ2Vγ9T cells in hepatocellular carcinoma [54]. NCRs, especially NKp46, which is negatively correlated with the risk of metastasis in colorectal cancer, are abundantly expressed on Vδ1T cells [58]. The ligand for NKp30 on Vδ1T cells is B7-H6, which is common in lymphoma and leukemia [59, 60] (Table 1).

In addition, tumor cells express a variety of specific surface proteins, which are of great significance to the functional activation of γδT cells (Table 1). The mitochondrial F1-ATPase-related structure has been detected on the surface of tumor cells. With binding to the delipidated form of apolipoprotein A-I (apo A-I), F1-ATPase shows the characteristic of being actively recognized by Vδ2Vγ9 TCR [61]. In recent years, annexin A2 was identified to be a ligand for Vδ3 TCR [62]. Annexin A2 is a phospholipid-binding protein in the cytoplasm and is exposed on the membrane in response to oxidative stress [63]. In general, tumor-specific surface proteins could constitute danger signals for γδT cells recognition, reducing the possibility of immune shielding.

Similar to natural killer cells, γδT cells can kill cancer cells indirectly by releasing abundant amounts of interferon-gamma (IFN-γ) thereby displaying a Th1 cell-like phenotype. γδT cells can also kill cancer cells directly via the death receptor signal factor associated suicide ligand (FasL) and TNF-related apoptosis-inducing ligand (TRAIL), secreting cytotoxic molecules such as granzyme and perforin [54]. Human MutS homologue 2 (hMSH2) is an ectopic nuclear protein associated with a variety of epithelial tumor cells. Both γδTCR and NKG2D participate in the recognition of hMSH2, stimulating the proliferation of Vδ2Vγ9T cells and enhancing IFN-γ mediated antitumor activity [64]. In addition, an increase in the number of Vδ1 T cells expressing CCR2, which produce IFN-γ, has been observed in melanoma and hepatocellular carcinoma [65, 66]. γδT cells with antigen presentation function (γδT-APCs) regulate CD4^+^ or CD8^+^ T cells, which initiate the adaptive immune response [67]. γδT-APC induces the mucosa to secrete calprotectin, which plays a role in the defense against intestinal mucosal inflammation [68].

#### Protumor effects (Fig. 2)

**Fig. 2:**
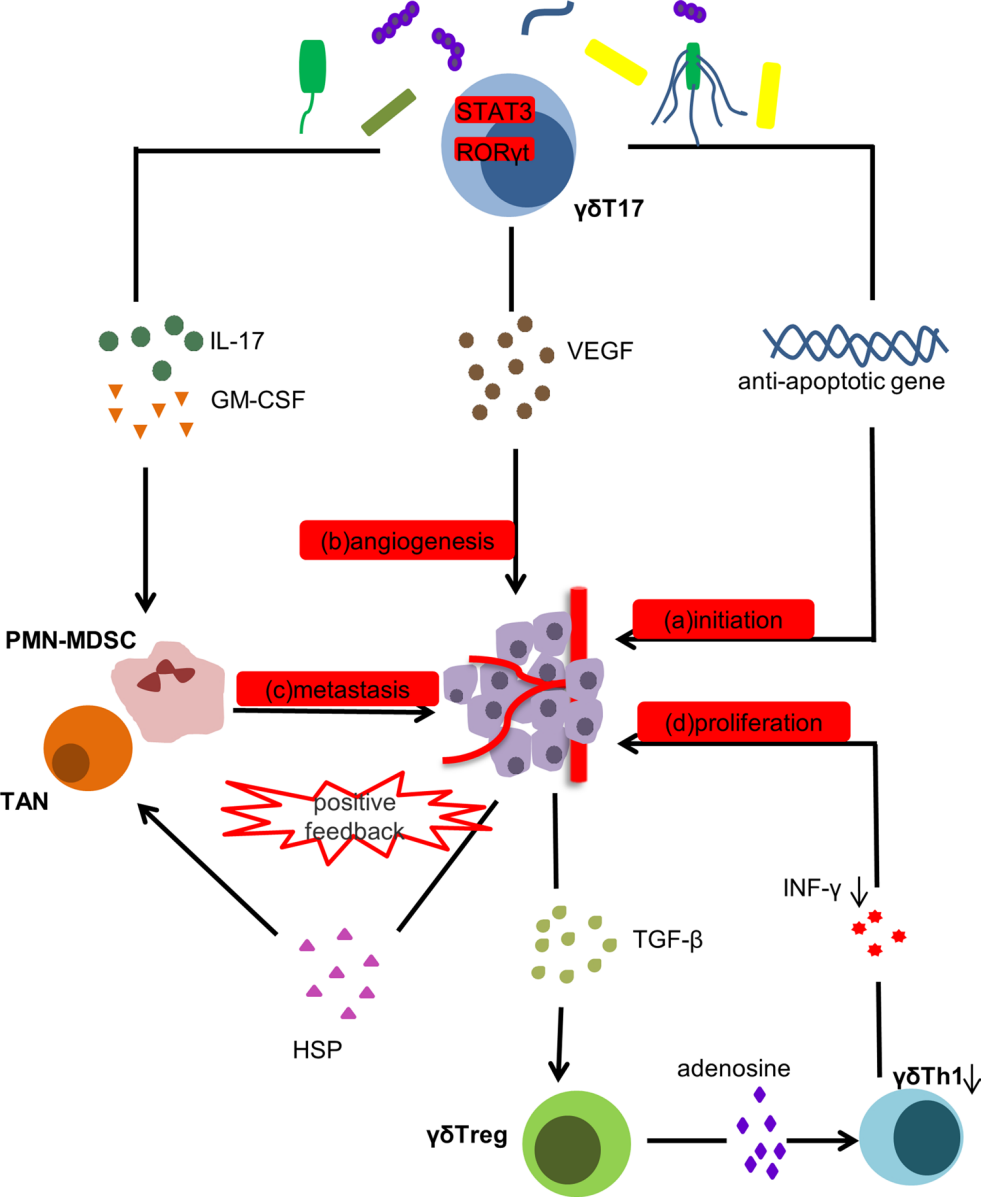
Protumor effects of γδT cells. γδT cells are activated and recruited to tumor sites under stimulation by commensal bacteria. Signal transducer and activator of transcription 3 (STAT3) and orphan nuclear receptor gamma t (RORγt) are essential transcription factors for γδT17 cells. a γδT17 cells express antiapoptotic genes, such as Bcl-2, McL-1 and Survivin, promoting the growth of cells with tumorigenic potential and imparting the possibility for tumor initiation. b γδT17 cells increase the concentration of vascular endothelial growth factor (VEGF), which promotes tumor angiogenesis. c Secretion of IL-17 is accompanied by production of granulocyte–macrophage colony-stimulating factor (GM-CSF), leading to accumulation of polymorphonuclear myeloid-derived suppressor cells (PMN-MDSCs) and tumor-associated neutrophils (TANs), which is conducive to tumor metastasis. PMN-MDSCs can also sense heat shock proteins (HSPs) released by cancer cells and play a further immunosuppressive role. d In addition, γδTregs can inhibit the secretion of interferon γ (INF-γ) from γδTh1 cells by increasing the amount of adenosine in the tumor microenvironment, resulting in excessive tumor proliferation

γδT cells exert unexpected protumor effects. The tumor-promoting functions are mainly due to IL-17-producing γδT cells. Current studies believe that tissue-resident Vδ1T cells are more inclined to differentiate into γδT17 cells, a finding seen in skin squamous cell carcinoma [69], colorectal cancer [70], breast cancer [71] and lung cancer [72]. Signal transducer and activator of transcription 3 (STAT3) is an indispensable transcription factor for IL-17 and it is also a target of antiapoptotic genes [73, 74]. The expression of genes such as Bcl-2, Mcl-1 and Survivin promotes the growth of cells with tumorigenic potential [74]. Secretion of IL-17 by γδT cells is accompanied by upregulation of the expression of granulocyte–macrophage colony-stimulating factor (GM-CSF), which leads to accumulation of polymorphonuclear myeloid-derived suppressor cells (PMN-MDSCs) [70] and tumor-associated neutrophils (TANs) [75] at tumor sites. Accumulation of PMN-MDSCs and TANs at tumor sites establishes an immunosuppressive network in the TME, such that local and distant tumor metastasis becomes possible [76]. Also, PMN-MDSCs respond to heat shock proteins (HSPs) in tumor exosomes to exert further immunosuppressive effects [77]. Furthermore, high expression of IL-17 by γδT cells is associated with a high microvessel density. IL-17 also increases the concentration of vascular endothelial growth factor (VEGF) to promote tumor angiogenesis and has a unique tumor-promoting effect in human colorectal [78] and gallbladder cancers [79].

The CD39^+^ γδT cells are a new type of γδTreg found in human colorectal cancer reported in 2020 by Hu et al*.* [80]. The phenotype of γδT cells is plastic, such that it is normal for different types of γδT cells to have functional crossover. CD39^+^ γδ T cells express high levels of FOXP3 and secrete IL-17 and GM-CSF. In addition to attracting PMN-MDSCs, CD39^+^ γδTregs can also inhibit the functions of γδTh1 cells by increasing the concentration of adenosine in the TME, which allows cancer cells to further escape immune attack. Unexpectedly, CD39^+^ γδT cells were found to exhibit more potent immunosuppressive activity than conventional CD4^+^ Tregs [80].

#### Clinical implication

At present, tumor therapy based on γδT cells has received increasing attention, as a satisfactory response has been achieved in combination with chemotherapy and immunotherapy. Zoledronate can upregulate the expression of isopentenyl pyrophosphate (IPP) in cancer cells [81]. Vδ2Vγ9T cells exposed to a large number of phosphoantigens can rapidly develop amplified antigen sensitivity and tumor recognition [82, 83]. Solid cancer cells pretreated with low concentrations of zoledronate can be quickly killed by Vδ2Vγ9T cells in vitro [84]. A combination of chemotherapeutic drugs, zoledronic acid and Vδ2Vγ9T cells has shown promising results in clinical trials [84]. In addition to Vδ2Vγ9T cells, research focused on Vδ1T cells has also showed promising results [85]. Afonso et al*.* in 2016 [86] defined a Vδ1-enriched (> 60%) and NKG2D-upexpressing cytotoxic cell type, namely DOT cells. By designing a two-step method with distinct IL-4 expansion and IL-15 differentiation stages, a large number of (> 2500-fold) DOT cells can be amplified in vitro to show special cytotoxicity against the MEC-1 cell of chronic lymphocytic leukemia, but not healthy autologous leukocytes [86].

Autologous chimeric antigen receptor (CAR)-T cell therapy has emerged as a star component of tumor immunotherapy in recent years. Specifically, CAR-T cell therapy has remarkable efficacy in the treatment of hematological tumors [87]. In spite of this success, CAR-T cell therapy based on αβT cells has not yet achieved a breakthrough in the treatment of solid tumors. The application space of CAR-T therapy is also limited by difficulty in applying the therapy in allogeneic cells. γδT cells make it possible to use allogenic CAR-T cells from donors due to their MHC-independent characteristics, and this method may be more convenient and economical than existing methods [88]. Based on this assumption, Utrecht University have validated CAR-T cells expressing given Vδ2Vγ9 TCR clone 5 (TEG001) in condition of a good manufacturing practice [89]. These heterozygous T cells are called T cells engineered with defined γδTCRs (TEGs) [90], and have undoubtedly brought light to this therapeutic idea. Corporation Lava is developing a type of bispecific antibody that connects cancer cells and γδT cells separately, improving the precision of targeting and prevents immune silencing of γδT cells [91]. In-depth research on butyrophilin has provided a very effective target for the development of small molecule drugs based on γδT cell therapy [92].

## Conclusion

Crosstalk between commensal bacteria and γδT cells increases the complexity and uncertainty of the tumor immune microenvironment. During the initiation of the tumor, γδT cells are triggered by bacteria and migrate to the effector sites. The function of the aggregated γδT cell population is further amplified, and γδT cells can directly kill tumor cells or indirectly inhibit tumor growth through receptor-ligand interactions. However, the presence of γδT17 cells is an unfavorable factor, and the immunosuppressive state created by these cells allows cancer cells to escape immune surveillance.

At present, there is no definite relationship between the structure and functional subsets of γδT cells. Both Vδ1 and Vδ2 γδT cells have potential use in immunotherapy against cancer. Reprogramming γδT cells to transform towards an antitumor phenotype through precise regulation is a hot research topic. As an impressive candidate for adoptive cellular therapy, γδT cells have broad therapeutic prospects.

## Data Availability

Not applicable.

## Abbreviations

AhRs: Aryl hydrocarbon receptors
apo A-I: Apolipoprotein A-I
BTN3A1: Butyrophilin 3A1
CAR: Chimeric antigen receptor
CCR: C–C motif chemokine receptor
CXCR: C-X-C chemokine receptor
DNAM-1: DNAX accessory molecule-1
*E. coli*: *Escherichia coli*
FasL: Factor associated suicide ligand
GF: Germ-free
GM-CSF: Granulocyte–macrophage colony-stimulating factor
HMBPP: (E)-4-hydroxy-3-methyl-but-2-enyl pyrophosphate
hMSH2: Human MutS homologue 2
HSPs: Heat shock proteins
ICAM-1: Intercellular adhesion molecule 1
IFN-γ: Interferon-gamma
IL: Interleukin
IPP: Isopentenyl pyrophosphate
MHC: Major histocompatibility complex
MICA/B: MHC class I-related chains A/B
MyD88: Myeloid differentiation factor 88
NCR: Natural cytotoxicity receptor
NKG2D: Natural killer, group 2, member D
PAMPs: Pathogen-associated molecular patterns
PMN-MDSCs: Polymorphonuclear myeloid-derived suppressor cells
RORγt: Acid-related orphan nuclear receptor gamma t
SPF: Specific pathogen-free
STAT3: Signal transducer and activator of transcription 3
TANs: Tumor-associated neutrophils
TGF: Transforming growth factors
TLRs: Toll-like receptors
TME: Tumor microenvironment
TNF-α: Tumor necrosis factor alpha
TRAIL: TNF-related apoptosis-inducing ligand
TRIF: TIR-domain-containing adaptor inducing interferon-β
ULBP: UL16-binding proteins
VAV1: Vav guanine nucleotide exchange factor 1
VEGF: Vascular endothelial growth factor
γδT-APCs: γδT cells with antigen presentation function
γδTh1: Helper γδT1
γδTreg: Regulatory γδT
